# Influence of the Thickness of the Overriding Plate on Convergence Zone Dynamics

**DOI:** 10.1029/2019GC008678

**Published:** 2020-02-14

**Authors:** Solenn Hertgen, Philippe Yamato, Benjamin Guillaume, Valentina Magni, Nicholas Schliffke, Jeroen van Hunen

**Affiliations:** ^1^ Univ Rennes, CNRS, Géosciences Rennes ‐ UMR 6118 Rennes France; ^2^ Institut Universitaire de France (IUF), Paris France; ^3^ The Centre for Earth Evolution and Dynamics, Department of Geosciences University of Oslo Oslo Norway; ^4^ Earth Sciences Durham University Durham UK

**Keywords:** convergence zone dynamics, overriding plate, rheology of the lithosphere, subduction/collision processes, mantle dynamics, 3D numerical modeling

## Abstract

The important role played by the upper plate in convergence zones dynamics has long been underestimated but is now more and more emphasized. However, the influence of its thickness and/or strength on orogenic systems evolution remains largely unknown. Here we present results from 3D thermo‐mechanical numerical simulations of convergence zones (including oceanic subduction followed by continental subduction/collision), in which we vary the rheological profile of the overriding plate (OP). For this, we systematically modify the crustal thickness of the overriding lithosphere and the temperature at the Moho to obtain a thermal thickness of the overriding lithosphere ranging from 80 to 180 km. While all models share a common global evolution (i.e., slab sinking, interaction between slab and the 660 km discontinuity, continental subduction/collision, and slab breakoff), they also highlight first‐order differences arising from the variations in the OP strength (thermal thickness). With a thin/weak OP, slab rollback is favored, the slab dip is low, the mantle flow above the slab is vigorous, and the trench migrates at a high rate compared to a thick/strong OP. In addition, slab breakoff and back‐arc basin formation events occur significantly earlier than in models involving a thick OP. Our models therefore highlight the major role played by the thickness/strength of the OP on convergence zone dynamics and illustrate its influence in a quantitative way.

## Key Points

- We present 3D thermo‐mechanical lithospheric‐scale models of oceanic subduction followed by continental subduction/collision
- We show that the overriding plate thickness strongly impacts the convergence zone dynamics and the kinematics of the subduction events
- Thicker overriding plate decreases mantle flow and trench mobility; it also controls overriding plate deformation and topography

## 1 Introduction

On Earth, convergence zones vary significantly with respect to the nature of the involved lithospheres (i.e., oceanic subduction vs continental subduction/collision), subduction modes (i.e., slab rollback vs slab advance), exhumed units, trench shapes, and deformation distributions. In particular, the overriding plate (OP) can have variable compositions, sizes, morphologies, thicknesses, thermal structures, and deformation patterns. Within the same tectonic context (e.g., continent‐continent convergence), slab dips, metamorphic rocks, plate boundary curvatures, deformation styles, or topographies can vary significantly. For instance, very localized deformation is observed in the Alps (Dewey et al., 1986), whereas deformation is distributed over thousands of kilometers through the Tibetan plateau (Beaumont et al., 2001, 2004; Clark & Royden, 2000; Dewey et al., 1986; Godin et al., 2006; Nábělek et al., 2009; Powell, 1986; Pusok & Kaus, 2015; Royden et al., 1997). On the other hand, convergent zones can share similar structures (e.g., orogenic plateaus in Tibet and in the Central Andes) despite their different tectonic settings (continental collision vs oceanic subduction, respectively).

### 1.1 Importance of OP in Subduction Zone Dynamics

Convergence zones have been the subject of many studies, in particular to unravel how and where the deformation develops and how it evolves over time. For this, numerous analog and numerical models have been performed. In some of these models that specifically focus on the dynamics of the subducting plate (SP), the OP is not implemented assuming that it would passively accompany the trench migration without affecting the mantle flow (e.g., Bellahsen et al., 2005; Christensen, 1996; Goes et al., 2008; Guillaume et al., 2010; Irvine & Schellart, 2012; Schellart, 2010 Stegman et al., 2006, 2010). Studies considering the SP in isolation show that slabs preferentially roll back (e.g., Funiciello et al., 2003, 2008; Kincaid & Olson, 1987; Morra et al., 2006; Schellart, 2004; Schellart et al., 2007; Stegman et al., 2006) and that the viscosity of the subducting slab relative to that of the surrounding mantle exerts a strong control on slab evolution, particularly the velocity at which the slab hinge retreats (e.g., Bellahsen et al., 2005; Di Giuseppe et al., 2008; Enns et al., 2005; Faccenna et al., 2007; Funiciello et al., 2003, 2008; Ribe, 2010). However, other studies have shown that the OP can have a major influence on the convergence zone dynamics (e.g., Arcay et al., 2008; Butterworth et al., 2012; Clark et al., 2008; Garel et al., 2014; Guillaume et al., 2009, 2018; Heuret et al., 2007; Lallemand et al., 2005; Meyer & Schellart, 2013; Rodríguez‐González et al., 2012; Van Dinther et al., 2010; Yamato et al., 2009). Indeed, as lithospheric material is stiffer and has a density contrast with the underlying upper mantle, the interactions between the SP and the OP therefore necessarily affect the mantle flow and the whole subduction dynamics. Hence, appreciating that subduction zones are coupled systems, most mantle‐scale models used to investigate the impact of the slab on the dynamics of convergence zones now include an OP (e.g., Becker et al., 1999; Magni et al., 2012; Toussaint et al., 2004; van Hunen & Allen, 2011). However, previous studies mainly focused on the SP and the role of the OP was often neglected.

### 1.2 Previous Studies on OP Dynamics

The OP has been studied both in isolation (e.g., Gautier et al., 1999; Hatzfeld et al., 1997; Martinod et al., 2000; Schellart et al., 2002) and through mantle‐scale models. Previous studies focused on the influence of the convergence dynamics on the deformation of the OP, looking in particular at the effects of the trench kinematics (Holt et al., 2015; Stegman et al., 2006), the SP properties (i.e., rheology and buoyancy), and the processes affecting the slab and the OP at depth (breakoff, underplating; e.g., Bottrill et al., 2012; Capitanio & Replumaz, 2013; Guillaume et al., 2013; Magni et al., 2014, 2017). Numerous physical parameters have been proposed to control the OP deformation:

- SP change of geometry (e.g., subduction of an aseismic ridge, slab tearing; Clark et al., 2008; Espurt et al., 2008; Martinod et al., 2013), slab age at trench (Molnar & Atwater, 1978; Salze et al., 2018);
- active diapirism in the mantle wedge under the back‐arc region (Karig, 1971);
- basal shear tractions resulting from subduction‐induced poloidal flow in the mantle wedge (e.g., Holt et al., 2015; Meyer & Schellart, 2013; Sleep & Toksöz, 1971; Toksöz & Hsui, 1978);
- shear coupling along the subduction zone interface (e.g., Lamb & Davis, 2003);
- slab rollback/advance and trench migration rate (e.g., Elsasser, 1971; Guillaume et al., 2009; Lonergan & White, 1997; Molnar & Atwater, 1978);
- convergence rate (e.g., Somoza, 1998).

These studies did not specifically focus on the role the OP and possible variations of its properties could have on the convergence dynamics. Other studies instead showed that the presence of an OP modifies the subduction dynamics by significantly reducing slab rollback velocities (e.g., Butterworth et al., 2012; Capitanio et al., 2010; Clark et al., 2008; Holt et al., 2015; Leng & Gurnis, 2011; Yamato et al., 2009). The absolute motion and changes in the velocity of the OP also appear to control slab geometry and OP deformation regime, with an OP accelerating toward the trench producing a flatter slab and promoting shortening (e.g., Carlson & Melia, 1984; Cerpa et al., 2018; Guillaume et al., 2018; Heuret et al., 2007). The geometry of the OP and its thickness in particular influence the slab dip angle (Meyer et al., 2013) and the trench retreat velocity (Sharples et al., 2014). The OP length and width also have an influence on the strain localization within the OP (Butterworth et al., 2012). Finally, the OP temperature profile influences the slab dip (Garel et al., 2014; Rodríguez‐González et al., 2012) and the rheology of the OP is also a key parameter as the crustal rheology impacts both the strain localization within the OP (Chen et al., 2017) and the whole subduction dynamics (e.g., Figure 11 in Yamato et al., 2008). A recent study also showed that depending on the employed rheological laws, slab morphology, trench migration rates, and topography of the OP may vary significantly (Pusok et al., 2018).

### 1.3 Motivations for This Study

Hence, the role played by the overriding lithosphere in convergent zones appears fundamental. However, its influence on the structure and time evolution of mountains belts and/or back‐arc basins at the surface has been incompletely addressed so far. In particular, the relationships between the OP thickness/strength and the slab dynamics, the plates/trench kinematics, that all control the OP deformation, remain to be explored. The aim of this study is therefore to perform 3D thermo‐mechanical numerical models to investigate how the initial OP rheological profile influences the convergence zone dynamics. For this, we tested a large range of possible thicknesses/strengths for the OP and show that it has a significant influence on the mode of subduction, the deformation pattern of the OP, the subduction kinematics, and the timing of strain localization both in the slab and in the OP. In addition, the temporal and spatial transition from oceanic subduction to continental collision also requires special attention in three dimensions as it is a common process in nature. This stimulated our choice of including a continental block as part of the SP in our 3D models. Furthermore, previous studies showed that collision of a buoyant indentor (such as a continental block) facilitates laterally OP extension and formation of a back‐arc basin due to the rotation of the oceanic part of the slab (Magni et al., 2014; Wallace et al., 2009). Moreover, an increase in the thickness of the OP leads to an increase in the length of the subduction interface, and the consequences on the coupling between the subducting and the OP may be important (e.g., Čížková & Bina, 2019; De Franco et al., 2006; Garel et al., 2014; Schmeling et al., 2008) and need to be studied.

## 2 Numerical Approach

### 2.1 Code Description

We designed 3D thermo‐mechanical numerical models of a convergence zone, in which oceanic subduction is followed by continental subduction and collision. Models were performed using the finite element code CITCOM (Moresi & Gurnis, 1996; van Hunen et al., 2005; Zhong et al., 2000). This code solves for conservation of mass, composition, momentum, and energy. As we consider an incompressible viscous medium and ignore density variations everywhere but in the driving force of the momentum equation (Boussinesq approximation), the conservation of mass is therefore described with a divergence‐free velocity field:

(1) $$\frac{\partial V_{x}}{\partial x}+\frac{\partial V_{y}}{\partial y}+\frac{\partial V_{z}}{\partial z}=0,$$

where *V*_*x*_ *, V*_*y*_ *,* and *V*_*z*_ correspond to the three components of the material velocity vector in the 3D (*x*, *y*, *z*) Cartesian coordinate system. The conservation of momentum is described as follows:

(2) $$-\frac{\partial P}{\partial x}+\frac{\partial \tau_{\mathrm{xx}}}{\partial x}+\frac{\partial \tau_{\mathrm{xy}}}{\partial y}+\frac{\partial \tau_{\mathrm{xz}}}{\partial z}=0$$

(3) $$-\frac{\partial P}{\partial y}+\frac{\partial \tau_{\mathrm{yy}}}{\partial y}+\frac{\partial \tau_{\mathrm{yx}}}{\partial x}+\frac{\partial \tau_{\mathrm{yz}}}{\partial z}=0$$

(4) $$-\frac{\partial P}{\partial z}+\frac{\partial \tau_{\mathrm{zz}}}{\partial z}+\frac{\partial \tau_{\mathrm{yz}}}{\partial y}+\frac{\partial \tau_{\mathrm{zx}}}{\partial x}=-\mathrm{\rho g}$$

and represents the balance among the pressure *P*, the deviatoric stress tensor *τ*_*ij*_, and the buoyancy forces acting in the system (with *ρ* and *g* that correspond to the density and the gravitational acceleration, respectively).

The relation between the stress tensor components and the velocity field is computed by using the constitutive relationship expressed as:

(5) $$\tau_{\mathrm{ij}}=\eta {\dot{\varepsilon }}_{\mathrm{ij}}$$

where *η* is the effective shear viscosity and ${\dot{\varepsilon }}_{\mathrm{ij}}$ is the strain rate tensor, defined as

(6) $${\dot{\varepsilon }}_{\mathrm{ij}}=\left(\frac{\partial V_{i}}{{\partial x}_{j}}+\frac{\partial V_{j}}{{\partial x}_{i}}\right).$$

The density is temperature (*Τ*) and composition (*C*) dependent and is computed from a reference mantle density *ρ*_0_ such that

(7) $$\rho =\rho_{0}-\alpha \rho_{0}\left(T-T_{0}\right)+\Delta \rho_{C}$$

where *α* is the thermal expansivity, *T*_0_ is the reference temperature (set to the surface temperature here), and *Δρ*_*C*_ is the density contrast between the mantle and the material considered (see Table 1). The conservation of energy is used to compute the temperature field evolution with time such that

(8) $$\frac{\partial T}{\partial t}+V_{x}\frac{\partial T}{\partial x}+V_{y}\frac{\partial T}{\partial y}+V_{z}\frac{\partial T}{\partial z}=\kappa \left(\frac{\partial^{2}T}{{\mathrm{dx}}^{2}}+\frac{\partial^{2}T}{{\mathrm{dy}}^{2}}+\frac{\partial^{2}T}{{\mathrm{dz}}^{2}}\right),$$

where *κ* is the thermal diffusivity (see Table 1). Note that, in this equation, the heat production rate is set to 0. At each time step, the compositional field, representing crust or mantle material, which influences local density and viscosity, is then advected by a particle‐tracing technique.

**Table 1:** Model Parameters, Symbols, and Units

Parameters and units	Symbol	Default value	Units
Viscous power law exponent	*n*	3.5(dis)/1 (dif)	‐
Dislocation creep prefactor	*A* ^*^	6.52 × 10^6^	Pa.s^1/n^
Diffusion creep prefactor	*B* ^*^	2.48 × 10^8^	Pa.s
Activation volume	*V*	0	m^3^.mol^−1^
Activation energy	*E* ^*^	360	kJ.mol^−1^
Gravitational acceleration	*g*	9.8	m.s^−2^
Gas constant	*R*	8.3	J·K^−1^·mol^−1^
Thermal diffusivity	*κ*	10^−6^	m^2^.s^−1^
Thermal expansion coefficient	*α*	3.5 × 10^−5^	K^−1^
Pressure	*P*	‐	Pa
Lithostatic pressure	*P*_0_	‐	Pa
Temperature	*T*	‐	°C
Surface temperature	*T*_surf_	0	°C
Asthenospheric temperature	*T*_asth_	1350	°C
Velocity (and components)	*u* (*V*_*x*_, *V*_*y*_, *V*_*z*_)		m.s^−1^
Compositional density contrast between the continental crust and the mantle material	*Δ* *ρ*_*C*_	−600	kg.m^−3^
Strain rate	$\dot{\varepsilon }$		s^−1^
Second invariant of the strain rate	${\dot{\varepsilon }}_{\mathrm{II}}$		s^−1^
Effective viscosity	*η*		Pa.s
Maximum viscosity	*η*_max_	10^23^	Pa.s
Friction coefficient	*μ*	0.1	
Reference density	*ρ*_0_	3,300	kg.m^−3^
Deviatoric stress	*Τ*	‐	MPa
Yield stress	*τ*_*y*_		MPa
Cohesion	*τ*_0_	40	MPa
Maximum yield stress	*τ*_max_	400	MPa
			
*Model geometry*			
Domain depth	*H*	660	km
Domain length	*L*	3,300	km
Domain width	*W*	3,960	km
Mesh resolution		from 8 × 8 × 8 to 20 × 20 × 20	km^3^
Continental block width	*‐*	1,320	km
Oceanic side width	*‐*	660	km
Continental crust thickness	*h*_c_	40	km
			
*Variables parameters*			
Moho temperature	*T*_Moho_		°C
Crust thickness	*h*_c_		km
Lithosphere thickness	*h*_l_		km

The materials constituting the model are considered as visco‐plastic. Such a visco‐plastic rheology combines a flow law for dislocation creep and diffusion creep, to simulate the viscous behavior of rocks, with a flow law simulating Byerlee's law (Byerlee, 1978) to account for their brittle feature (van Hunen & Allen, 2011).

Considering the viscous part, both dislocation creep law and diffusion creep law are defined as

(9) $${\dot{\varepsilon }}_{\mathrm{II}}=A{\tau_{\mathrm{II}}}^{n}\exp\left(-\frac{E+\mathrm{PV}}{\mathrm{RT}}\right),$$

where ${\dot{\varepsilon }}_{\mathrm{II}}$, *τ*_II_, *n*, *A, E, P, V*, and *R* correspond to the second invariant of strain rate, second invariant of deviatoric stress, the power law exponent, the power law preexponent, the activation energy, the pressure, the activation volume, and the gas constant, respectively (see Table 1).

By using this formulation, a viscosity related to dislocation creep *η*_dis_ can then be defined as

(10) $$\eta_{\mathrm{dis}}=\frac{\tau_{\mathrm{II}}}{{\dot{\varepsilon }}_{\mathrm{II}}}=A^{-1/n}{{\dot{\varepsilon }}_{\mathrm{II}}}^{\frac{1-n}{n}}\exp\left(\frac{E+\mathrm{PV}}{\mathrm{nRT}}\right),$$

which can be simplified to

(11) $$\eta_{\mathrm{dis}}=A^{*}{{\dot{\varepsilon }}_{\mathrm{II}}}^{\frac{1-n}{n}}\exp\left(\frac{E^{*}}{\mathrm{nRT}}\right),$$

considering an activation volume equal to zero and that the prefactor *A* ^*^ is expressed in Pa.s^1/n^ (see Table 1).

In the same manner, a viscosity related to the diffusion creep can be expressed such as

(12) $$\eta_{\mathrm{dif}}=B^{*}\exp\left(\frac{E^{*}}{\mathrm{RT}}\right),$$

where *B* ^*^ is expressed in Pa.s, with *n* = 1 and by assuming a constant grain size.

The values used in our model for *n*, *E* ^*^, *A* ^*^, and *B* ^*^ are provided in Table 1. The chosen values do not directly correspond to precise experimental data but are in the range of the classical published values (e.g., Hirth & Kohlstedt, 2003; Karato & Wu, 1993; Korenaga & Karato, 2008). Instead, these values allow to model the dominance of diffusion/dislocation creep observed from seismic anisotropy patterns (i.e., dislocation creep for shallow upper mantle and in/around lithosphere/slabs, and diffusion creep for deeper upper mantle and away from vigorous convection). The obtained values for absolute viscosity also fit estimates for upper mantle values (~10^20^ Pa.s) from postglacial rebound studies (e.g., Lambeck et al., 1998) and geoid estimates (e.g., Hager, 1991). In our models, a composite viscosity *η*_*v*_ is then computed from equations (11) and (12), such as

(13) $$\eta_{v}=\min\left(\eta_{\mathrm{dis}},\eta_{\mathrm{dif}}\right).$$

The brittle behavior is simulated by computing an apparent viscosity defined as

(14) $$\eta_{p}=\frac{\tau_{y}}{\dot{\varepsilon }}$$

where *τ*_*y*_ is the yield stress described as

(15) $$\tau_{y}=\min\left(\tau_{0}+\mu P_{0},\tau_{\max}\right)$$

where *τ*_max_ is the maximum yield stress (set to 400 MPa) and *τ*_0_+*μP*_0_ is a depth‐dependent von Mises model (Spiegelman et al., 2016), where *τ*_0_ and *μ* are the cohesion and the friction coefficients, respectively, and *P*_0_ is the lithostatic pressure. In each point of the model, the effective viscosity *η* corresponds to the minimum of viscosity values derived from each mechanism (i.e., *η* = min(*η*_v_, *η*_p_)). The resulting viscosity is however limited by the maximum viscosity *η*_max_ (set to 10^23^ Pa.s) as the effective viscosity contrast between lithosphere and mantle is typically thought to be 2–3 orders of magnitude (see Goes et al., 2011 and references therein).

### 2.2 Model Setup

**Figure 1:**
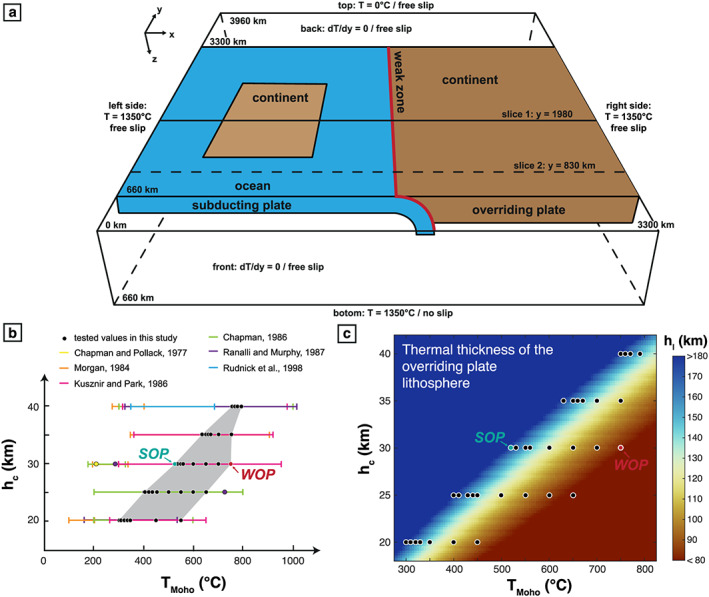
(a) Model setup showing the initial geometry of the model and presenting the thermal and mechanical boundary conditions: blue refers to oceanic domains and brown to continental domains. (b) Initial continental overriding plate Moho temperatures (*T*_Moho_) and crustal thicknesses (*h*_c_) tested in this study (black circles). Maximum and minimum values of Moho temperatures versus depths in natural cases from previous studies are also shown for comparison. The two highlighted models (WOP, thin/weak overriding plate and SOP, thick/strong overriding plate) correspond to the models described in details in the text. (c) Thermal thickness of the overriding plate lithosphere (*h*_l_) as a function of *h*_c_ and *T*_Moho_ (see text for details). Black circles correspond to the models used in this study. The two highlighted models (WOP and SOP) are also indicated.

The initial model setup is presented in Figure 1a and closely follows the one used by Magni et al. (2014). The dimensions of the box are 3300 km (length, *x* axis), 3960 km (width, *y* axis), and 660 km (depth, *z* axis), with a spatial mesh resolution from 8 × 8 × 8 km^3^ around the slab to 20 × 20 × 20 km^3^ deeper in the model box. The model involves both a homogeneous continental OP and an oceanic SP including a continental block placed at the center of the subduction zone (Figure 1a). In that way, the continental block reaches the trench after 500 km of oceanic subduction. In order to initiate subduction without imposing any external forces, the model starts with a 200 km‐long slab. The dip of the initial oceanic subduction is constrained by the imposed initial curvature radius of 500 km and is then free to evolve. The initial position of the trench is set to *x* = 1,650 km. The position of the trench is then also free to move during the model evolution (Magni et al., 2012). The OP and the SP are separated by a mobile weak zone (Magni et al., 2012), that is, a zone constituted by a low‐viscosity material with upper mantle rheology, which facilitates the subduction processes. The effect on the plate coupling of the introduction of such a weak zone in our model is discussed in section 6. To allow mantle flow around the edges of the slab, the subducting and the overriding plates are adjacent to oceanic lithospheres in which subduction does not occur. This is modeled by imposing two transform faults simulated by two low‐viscosity zones (10^20^ Pa.s) of 20 km width at *y* = 660 km and *y* = 3,300 km, respectively (Magni et al., 2014; van Hunen & Allen, 2011). The initial temperature field for the oceanic lithosphere is calculated following a half‐space cooling solution for a 50‐My‐old plate (Turcotte & Schubert, 2002), which corresponds to a ~80‐km thick lithosphere. The reference density in the model is 3300 kg/m^3^. The continental crust is modeled with a layer of positively buoyant crust with a density contrast of 600 kg/m^3^. In the continental block that is embedded in the SP, the initial temperature decreases linearly from the surface temperature (set to 0 °C) to a temperature equal to 1350 °C at 150 km. Thermal boundary conditions (Figure 1a) are fixed at the top and the bottom of the model box and set to *T* = 0 °C and *T* = 1350 °C, respectively. The left and right sides are also fixed to the temperature of the asthenosphere such as *T* = *T*_asth._ = 1350 °C (see Table 1). The other boundaries are fully insulating. Mechanical boundary conditions are free slip everywhere, except at the bottom boundary of the model box where a no‐slip condition is applied to model the effect of a viscosity contrast between the upper and lower mantle (Figure 1a). Because there is no free surface on top of the model, the topography is evaluated from the vertical stresses at the surface such that

(16) $$h=\frac{\tau_{\mathrm{zz}}}{\rho_{s}g},$$

where *ρ*_s_ corresponds to the local density. The absolute elevation is then calibrated by considering a depth of −2.5 km at the mid‐ocean ridge. The model does not include erosion or sedimentation.

In our models, the strength of the overriding lithosphere is controlled by the thermal profile imposed at the beginning of the experiment. This initial thermal profile is defined by the temperature at the Moho (*T*_Moho_) and the crustal thickness (*h*_c_) of the continental lithosphere. This geotherm is first computed independently, by taking into account both the crustal thickness (*h*_c_) and the temperature at the Moho (*T*_Moho_). To do so, the heat equation (including heat diffusion and radiogenic heat production for the crust) is solved vertically in 1D for different lithospheric thermal thicknesses (*h*_l_) until reaching the steady state. The thermal properties for the crust and the mantle are as for the 3D models (see Table 1). Boundary conditions are also the same (i.e., *T*_surf_ = 0 °C at the surface and *T*_asth_ = 1350 °C for the adiabatic asthenosphere. We then extract the thermal thickness of the lithosphere (*h*_l_) that produces the desired temperature at the Moho. Hence, the thermal thickness of the lithosphere is directly related to both the thickness of the crust and the temperature at the Moho (Figure 1c). Depending on *T*_Moho_ and *h*_c_, the initial OP yield strength envelop is therefore different. As a consequence, a stronger lithosphere also corresponds to a thicker thermal lithosphere (*h*_l_).

To investigate the influence of the OP thickness/strength on the subduction dynamics, we carried out a parametric study for the following two parameters: *T*_Moho_ and *h*_c_. We systematically modified the crustal thickness of the overriding lithosphere from 20 to 40 km and tested different temperatures at the Moho ranging between 300 and 800 °C (Figure 1b, black dots). These values are within the range of natural values proposed by studies focused on the heat flux for the continental lithosphere (e.g., Chapman, 1986; Chapman & Pollack, 1977; Kusznir & Park, 1986; Morgan, 1984; Ranalli & Murphy, 1987; Rudnick et al., 1998; see Figure 1b). They correspond to thermal thicknesses (defined here as the depth where the temperature reaches 1350 °C) for the overriding lithosphere ranging from 80 to 180 km (Figure 1c). Knowing the thermal lithospheric thickness (*h*_*l*_) corresponding to a given temperature/depth of the Moho, we establish the initial temperature in the OP (*T*_OP_) as a function of depth (*z*) in the models by using the following equations:

(17) $$T_{\mathrm{OP}}\left(z\right)=T_{\text{surf}}+\left(\frac{z}{h_{c}}\right)\times \left(T_{\text{Moho}}-T_{\text{surf}}\right),\;\text{for}\;z<\mathrm{hc}$$

(18) $$T_{\mathrm{OP}}\left(z\right)=T_{\text{Moho}}+\left(\frac{z-h_{c}}{h_{l}-h_{c}}\right)\times \left(T_{\text{asth}}-T_{\text{Moho}}\right),\;\text{for}\;hc<z<hl$$

where *T*_surf_, *T*_Moho_, and *T*_asth_ correspond to the temperatures at the surface of the OP, at the Moho, and in the asthenosphere, respectively; *h*_c_ and *h*_l_ are the crustal and lithospheric thermal thicknesses.

## 3 Common Evolution of All Models

**Figure 2:**
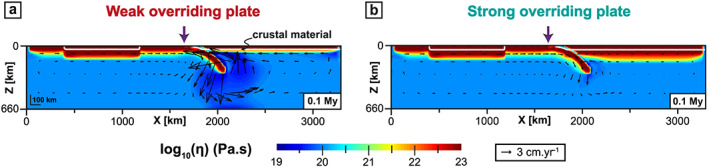
Effective viscosity field along *x*‐*z* cross sections in the center of the subduction zone (*y* = 1,980 km, see solid line in Figure 1a) when the slab starts sinking (0.1 My) for (a) a weak overriding plate (WOP) and (b) a strong overriding plate (SOP). Purple arrows indicate the location of the trench. Black arrows correspond to the mantle velocity field.

**Figure 3:**
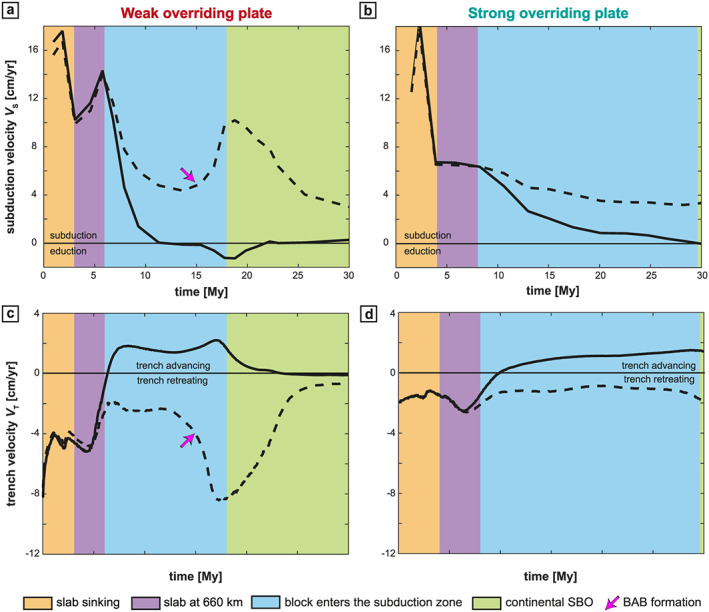
Time evolution of subduction and trench kinematics for the WOP (left) and the SOP (right) models. (a and b) Subduction velocity computed as *V*_*S*_ = *V*_SP_ − *V*_T_ where *V*_SP_ correspond to the SP absolute velocity and *V*_T_ to the trench velocity. Velocities are extracted both in the middle of the subduction zone (*y* = 1,980 km, solid line) and in the middle of the oceanic unit (*y* = 830 km; dashed line). Positive values indicate subduction, and negative values indicate eduction. (c and d) Trench velocity computed in the *x* direction for the same transects. Positive values indicate a trench moving toward the OP (prograde motion), negative values correspond to a trench motion toward the SP (retrograde motion). The different colors indicate the four different subduction stages described in the text. Orange: slab sinking; violet: the slab tip interacts with the 660‐km depth discontinuity; blue: the continental block enters the subduction zone; green: the slab breaks off. (SBO = slab breakoff; BAB = back‐arc basins reaching the profile).

**Figure 4:**
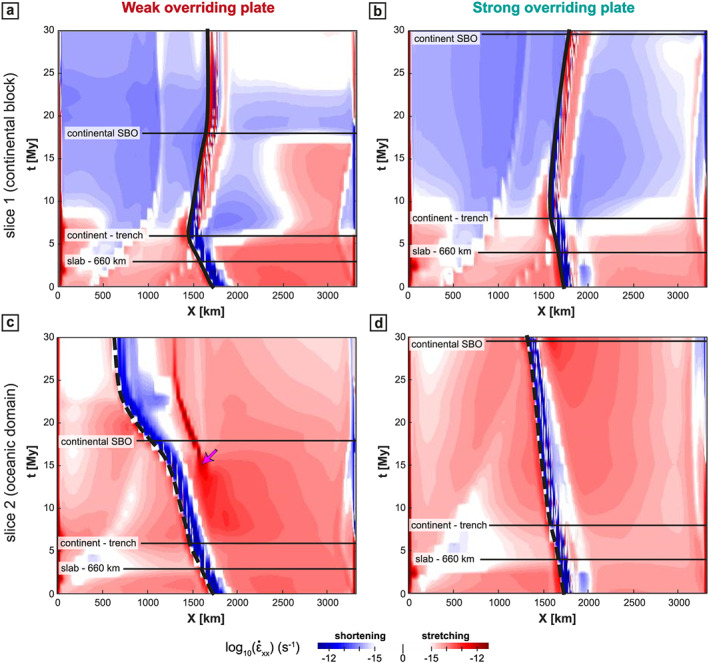
Time evolution of the horizontal strain rate component along the *x* axis ( $\dot{Ɛ}$_*xx*_) at the surface for the WOP (left) and SOP (right) models. (a and b) Profiles are taken from the middle of the subduction zone (*y* = 1,980 km; solid line in Figure 1a). (c and d) Profiles are taken from the middle of the oceanic domain (*y* = 830 km; dashed line in Figure 1a). Red indicates that OP is undergoing stretching, and blue corresponds to shortening. The thick black line indicates the trench position. The limits between the four main stages (see Figure 3) are indicated with thin horizontal black lines.

**Figure 5:**
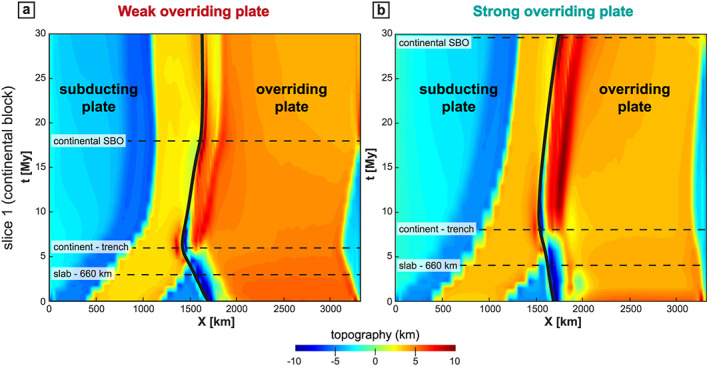
Time evolution of the topography at the latitude of the continental block (*y* = 1980 km, solid line in Figure 1a) for (a) the WOP model and (b) the SOP model. Black line indicates the trench position. The limits between the four main stages (see Figure 3) are indicated with thin horizontal dashed lines. The topography is computed from the vertical stresses, which constitute a proxy to evaluate the topography in models without free surface (and by considering a depth of −2.5 km at the mid‐ocean ridge).

**Figure 6:**
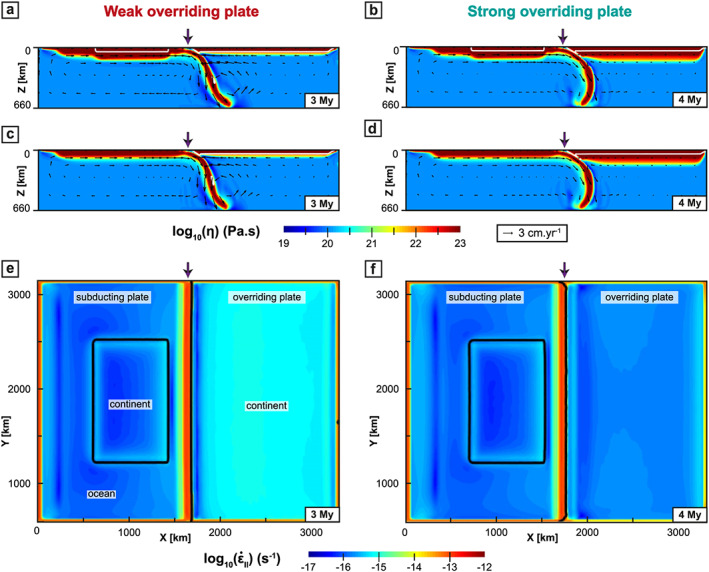
Cross sections showing the WOP (left) and SOP (right) models when the tip of the slab reaches the 660‐km depth discontinuity. (a–d) The *x*‐*z* cross sections presenting the viscosity field from (Figures 6a and 6b) the center of the subduction zone (*y* = 1980 km, see Figure 1a) and (Figures 6c and 6d) the latitude of oceanic subduction (*y* = 830 km, see Figure 1a). (e and f) The *x*‐*y* cross sections of the second invariant of the strain rate at the surface of the model (other legends are identical to Figure 2).

**Figure 7:**
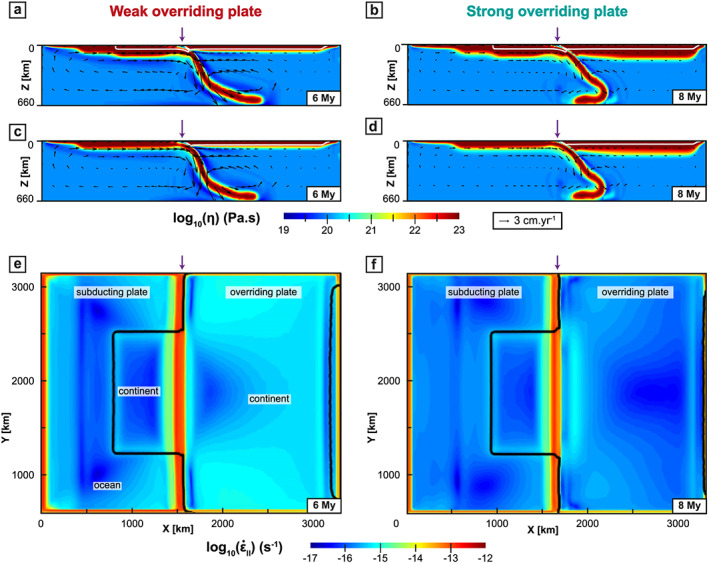
Cross sections showing the WOP (left) and SOP (right) models when the continental block arrives at the trench (legends are identical to Figure 2).

**Figure 8:**
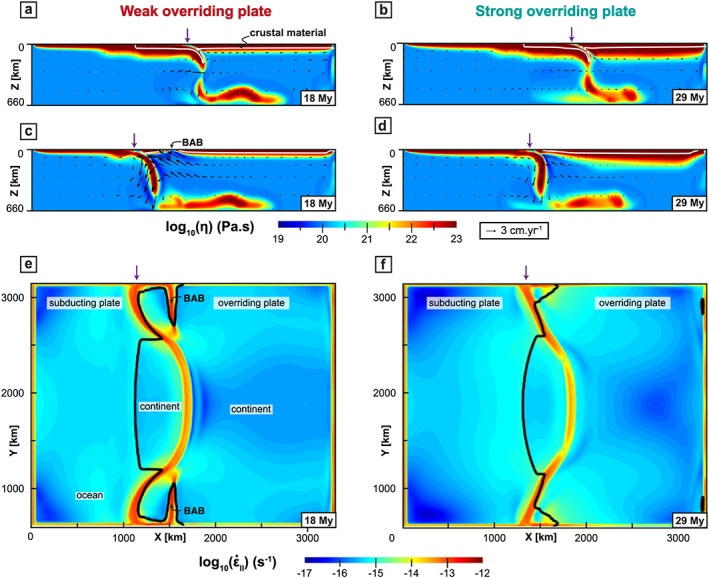
Cross sections showing the WOP (left) and SOP (right) models when the slab breaks off at the latitude of the continental block (legends are identical to Figure 2).

In all experiments, the subduction process evolves in four stages that can be summarized as follows (see also Magni et al., 2012):

1. Stage 1: The slab sinks into the upper mantle (Figure 2). The subduction velocity (*V*_S_) increases as the amount of SP entering the asthenosphere increases (Figures 3a and 3b). *V*_S_ then decreases when the leading edge of the slab approaches the upper‐lower mantle interface (Figures 3a and 3b). During this stage, the trench retreats toward the SP (Figures 3c and 3d) and is accompanied by stretching of the OP, apart from the arc area (300 km closest to the trench) where shortening develops (Figure 4). Close to the trench, the topography of the OP is low (Figure 5) and corresponds to the negative dynamic topography due to the slab sinking.
2. Stage 2: The slab interacts with the 660‐km‐depth upper‐lower mantle interface (Figure 6). The trench velocity (*V*_T_) then increases (Figure 3, purple area) and the OP still accommodates the trench retreat by stretching, except for the first 300 km from the trench (Figure 4).
3. Stage 3: The continental block enters the trench at the center of the subduction zone (Figure 7). Continental subduction is active until the leading edge of the continental block reaches depths of around 300 km. During this stage, important variations along the convergence zone start developing. At the latitude of the continental block (slice 1 in Figure 1a), *V*_S_ drastically slows down due to the decrease of slab pull associated with the low density of the crust within the continental block (Figures 3a and 3b, blue area). The trench starts to advance (Figures 3c and 3d) leading to the shortening of the back‐arc region of the OP (Figures 4a and 4b). Laterally, in the oceanic domains, subduction is still active and the trench keeps retreating but at rates corresponding to about half of their value prior to the arrival of the continental block (Figures 3c and 3d). It induces a continuous stretching of the OP (Figures 4c and 4d) with strain rates higher than 10^−14^ s^−1^. The opposite trench motion between continental and oceanic parts causes a concave curvature of the trench toward the SP (Figure 8).
4. Stage 4: At the latitude of the continental block, the slab starts to break off by a necking process localized at the continent/ocean boundary at ~300 km depth (Figure 8) because of the interaction of two opposite forces: the negative buoyancy associated with oceanic lithosphere subduction at depth and the positive buoyancy forces resulting from the low density continental crust. It leads to the opening of a slab window starting at the center of the continental block and propagating horizontally toward its edges (as already shown in van Hunen & Allen, 2011).

While these four stages are shared among all models, first‐order differences also arise from the imposed initial thermal profile. In the following, we discuss these differences by focusing on two end‐members that share the same crustal thickness *h*_crust_ = 30 km (Figure 1b). The first model is a “weak” end‐member model (i.e., a thin and hot OP) named hereafter “WOP” and characterized by a temperature at the Moho (*T*_Moho_) of 750 °C and a lithosphere thickness (*h*_lith_) of 80 km (see Figure 1b). The second model is a “strong” end‐member (i.e., involving a thick and cold OP) named hereafter “SOP” and characterized by *T*_Moho_ = 520 °C and *h*_lith_ = 180 km (see Figure 1b).

## 4 Weak Versus Strong OP Models

In this section, the four stages previously mentioned are described with emphasis on the differences between the two end‐members.

### 4.1 Slab Sinking

In the model with a WOP, the slab sinking into the upper mantle is accompanied by a counterclockwise poloidal flow that develops above the slab and brings low‐viscosity material into the mantle wedge (Figure 2a). In the model with a SOP, the mantle flow is less intense beneath the OP (Figure 2b). During the first step of oceanic subduction, *V*_T_ is almost 2 to 4 times smaller than in the WOP model (Figure 3). The OP displays lower strain rates in extension both in front of the continental and oceanic parts (Figure 4) and lower topography (Figure 5).

### 4.2 Interaction Between the Slab and the 660‐km Depth Discontinuity

In the WOP model (Figure 6, left panel), the slab reaches the 660‐km depth discontinuity after 3 My with a shallow slab dip *α*_s_ (slab dip measured at 100‐km depth) of ~50° and a deep slab dip *α*_d_ (slab dip at 300‐km depth) of ~70°. The poloidal mantle flow evolves into two cylindrical cells, a wide one (~2000 km) beneath the SP with a clockwise motion and a narrower one (~500 km) beneath the OP with a counterclockwise motion (Figures 6a–6c). Subduction occurs under slab rollback associated with a fast trench retreat (*V*_T_ > 4 cm/year; Figure 3c) leading to stretching in the OP (Figure 4a–4c). Within the OP, strain rate is relatively homogeneous (10^−15^ s^−1^, Figure 6e). High elevations (>3 km) are distributed across the whole OP, apart from the first 300 km from the trench (Figure 5a).

In models with a SOP (Figure 6, right panel), the slab reaches the 660‐km depth 1 My later (after 4 My), with a lower *α*_s_ (~45°) and a higher *α*_d_ (~80°) ultimately resulting in a forward bending of the slab. The mantle flow only displays a single large clockwise convection cell beneath the SP, which, along with a limited mantle flow toward the slab beneath the OP, favors slab folding (Figures 6b–6d). *V*_T_ is lower than in the WOP case (~2 cm/year; Figure 3c). The interior of the OP mainly deforms under trench‐perpendicular stretching with a decrease of ${\dot{\varepsilon }}_{\mathrm{xx}}$ from values of ~10^−14^ s^−1^ to values below 10^−15^ s^−1^ (Figures 4b–4d). Within the OP, strain rates are very low (~10^−15^–10^−16^ s^−1^; Figure 6f). Elevations in the center of the convergence zone are on average ~2 km, around 1 km lower than in the weak model (Figure 5b).

### 4.3 Continental Subduction

For the WOP model (Figure 7, left panel), the continental block arrives at the trench 6 My after the beginning of the model. *α*_s_ is then ~65° and *α*_d_ ~60°. Beneath the OP, the poloidal mantle flow forms a counterclockwise cylindrical cell over ~1,500 km, extending from the slab to the middle of the OP in the *x* direction (Figures 7a–7c). In front of the continental block, the deformation switches from stretching to shortening in the frontal part of the OP but still undergoes stretching at the back (Figure 4a). The deformation is quite homogeneous through the OP; the strain rates are on average higher in front of the oceanic slab (~ 10^−15^ s^−1^) than at the front of the continental block (10^−16^ s^−1^, Figure 7e). The topography in front of the continental block is smooth with values of 7–8 km in the first 150 km on the edge of the OP and values of ~5 km in the rest of the OP (Figure 5a).

For the SOP model (Figure 7, right panel), the continent arrives at the trench 2 My later than in the WOP experiment (at 8 My) and with a lower *α*_s_ ~45° and *α*_d_ ~35°. At depth, the slab displays a folded shape all along the subduction zone (Figures 7b–7d). Beneath the OP, the mantle flow forms a counterclockwise cell restricted to ~500 km in the *x* direction (Figures 7b–7d). In front of the continental block, the deformation mode switches from stretching to shortening in the entire OP (Figure 4b). The highest strain rates (10^−15^ s^−1^) are found around 150 km inland of the OP in front of the continental block (Figure 7f) where the surface reaches up to 10 km in elevation while it decreases down to 4 km in the rest of the OP (Figure 5b).

In the oceanic subduction domain, the WOP stretches and thins to accommodate the slab retreat, ${\dot{\varepsilon }}_{\mathrm{xx}}$ reaches values of up to ~10^−12^ s^−1^, and at ~11 My, back‐arc basins start forming in the OP at ~400 km from the trench. The time for the back‐arc formation is defined in this study when OP deforms under extension with a strain rate increase by one order of magnitude in comparison with the first stages (i.e., over 10^−14^ s^−1^ in this case). Back‐arc basin formation starts at the edge of the model and propagates laterally, which explains why it is only visible at ~14–15 My in Figure 4c that corresponds to a section located 170 km inside the model. Back‐arc basins are then best seen in Figure 8e taken 7 My later. After a few My, the mantle indeed flows upward toward the zone of the back‐arc basins, which isolates a proximal block of the OP that eventually rotates toward the continental block (Figure 8e).

For the SOP model, the amount and velocity of trench retreat for the oceanic parts are about half that of the WOP model (Figures 3c and 3d), which eventually results in a less curved trench (Figure 8f). In front of the oceanic domains, the maximum deformation is located in the frontal part of the OP, which is stretched to accommodate the retreat of the slab. At 29 My, an extensional event leading to a thinner lithosphere (close to but much less localized than a back‐arc basin in a strict sense) occurs at ~250 km from the trench (*x* = 1550 km, Figure 8f). The mantle then flows up to the front of the OP and participates to the localization of the stretching.

### 4.4 Slab Breakoff

In the WOP experiment, the slab breakoff takes place in the center of the subduction zone at 18 My (Figure 8a), that is, 12 My after the initiation of the continental block subduction. The breakoff then propagates laterally in the *y* direction, leading to the formation of a slab window, through which mantle material is channeled (in agreement with van Hunen & Allen, 2011). At the latitude of the continental block, the buoyant continental material is exhumed by eduction for ~5 My (Figure 3a). At the same time, the OP deforms under shortening. Significant shortening ceases as the trench stops advancing after ~23 My (Figures 3c and 4a). The elevation decreases in the frontal part of the OP to values as low as ~1 km but maintains values around 5 km in the rest of the OP (Figure 5a).

Beneath the SOP, the slab breakoff occurs later at 29 My (Figure 8b), that is, 21 My after the continental collision (i.e., 10 My later in comparison with the WOP). In this experiment, at the latitude of the continental block, no eduction phase is recorded during slab breakoff. The trench still advances at *V*_T_ = 1.5 cm/year (Figure 3d), which further promotes shortening of the OP (Figure 4b).

### 4.5 End of the Model (From Breakoff to 30 My)

Between continental subduction and the end of the model calculation at 30 My, the trench acquires a curved shape for both the WOP and SOP models. This curvature is more pronounced after 30 My for the WOP model, with a maximum amount of absolute trench advance of ~210 km at the latitude of the continental block subduction (slice 1) and a maximum amount of absolute trench retreat of ~1100 km at the latitude of oceanic subduction (slice 2; Figures 4a–4c). For the SOP model, after 30 My, the trench is less curved. If the center of the convergence zone displays a maximum absolute trench advance of ~210 km, similar to the WOP model, the edges instead only experienced a maximum absolute trench retreat of ~320 km (Figures 4b–4d).

At 30 My, at the latitude of the continental block, the WOP does not deform any more while the SOP still deforms under shortening (Figures 4a and 4b). Significant deformation is thus recorded for a longer period of time for the SOP model (> 20 My) than for the WOP model (< 20 My). Moreover, the frontal part of the OP (the first 200 km from the trench) shows a clear discrepancy between elevations lower than the rest of the OP for the WOP and elevations higher than the rest of the OP for the SOP model (Figure 5).

## 5 Impact of the Initial Thermal Profile

The two end‐members described above clearly highlight differences in subduction dynamics associated with the strength of the OP as defined by a different initial thermal profile. To better study what controls these different behaviors and how the transition between these two end‐members occurs, we performed a set of 32 models. All these models have the same setup, but we systematically varied both the temperature at the Moho from 300 to 800 °C and the depth of the Moho within the range 20–40 km (Figure 1b). This allows us to quantify the effect of the OP rheology on the subduction dynamics. Indeed, the hotter/thinner the *T*_Moho_/thickness of the crust is, the weaker is the OP lithosphere.

### 5.1 Effect on the Subduction Mode

**Figure 9:**
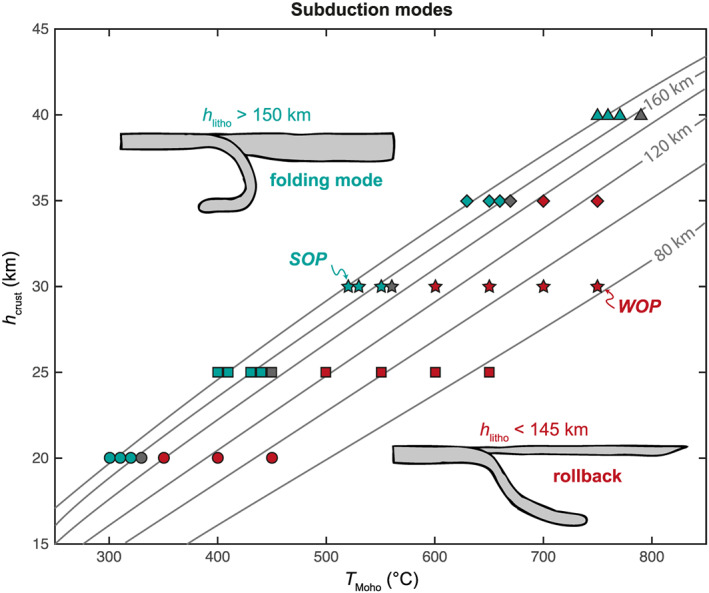
Subduction modes as a function of the crustal thickness and temperature at the Moho. Red symbols indicate slab rollback, blue symbols slab forward folding, and gray symbols the intermediate mode. Gray lines indicate the interpolated corresponding lithospheric thermal thickness (*h*_l_, see Figure 1c).

With the two end‐members described previously, we show two principal modes of subduction: slab rollback for the WOP and slab forward folding for the SOP. This trend is confirmed by the entire set of models included in our parametric study (see Figure 9). Results are in line with the results from 2D numerical models carried out by Garel et al. (2014) and show that thin and “hot” OP leads to slab rollback, while a thick and “cold” OP leads to slab forward folding. The transition between these two modes is quite sharp and occurs at Moho temperatures of ~330 °C, ~560 °C, and ~790 °C for a crustal thickness of 20 km, 30 km, and 40 km, respectively (Figure 9). The corresponding crustal thermal gradients (*T*_Moho_/*z*_Moho_) range between ~15 and ~20 °C/km and define an initial thermal lithospheric thickness (i.e., depth of the 1350 °C isotherm) of ~150 km. At this transition, a third mode of subduction is observed showing slab rollback at shallow depths and slab forward folding at the upper‐lower mantle transition zone.

### 5.2 Effect on the Timing of Strain Localization in the Slab and OP

**Figure 10:**
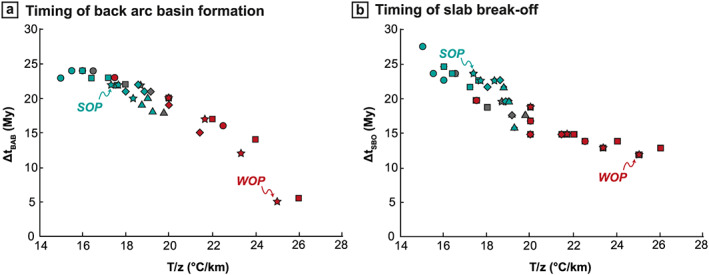
(a) Timing of back‐arc basins formation after continental subduction initiation as a function of the mean crustal thermal gradient (colors and symbols are the same as in Figure 9). (b) Timing of slab breakoff after continental subduction initiation.

A change in the initial thermal profile of the OP also drastically impacts the timing of back‐arc basin formation in front of the oceanic domains. Figure 10a shows the time required for the back‐arc basins (or for the significant thinning of the lithosphere, see section 4.3.) to form after the onset of continental subduction (Δ*t*_BAB_) as a function of the initial crustal thermal gradient. Results presented in Figure 10a show that the higher the OP strength (low thermal gradient) is, the longer the time required to open the back‐arc basins is, from 5 My after continental subduction initiation for the weakest OP to ~25 My for the strongest OP. For a crustal thermal gradient lower than 16 °C/km, the timing of the back‐arc basin formation seems to stabilize at ~23–25 My.

The OP initial thermal profile impacts the OP deformation itself and also the timing of the SP deformation (Figure 10b). The time required for the slab to break off after the initiation of continental subduction (Δ*t*_SBO_) varies from a minimum value of 11 My for the weakest OP (initial *T*_Moho_ */h*_c_ ≈ 25 °C/km) to values up to 28 My for the strongest OP (initial *T*_Moho_ */h*_c_ ≈ 15 °C/km). The relationship between *t*_SBO_ and *T*_Moho_ */h*_c_ is not linear but rather tends toward an asymptote as *T*_Moho_ */h*_c_ increases. As the slab breakoff depth is independent of the OP rheology and always occurs at the same depth of ~300 km, the timing of breakoff is controlled by the time required for the continental block leading edge to reach this depth, which depends on the subduction velocity. For instance, after the arrival at the trench of the continental block and before slab breakoff, the average subduction velocity for the WOP model at this latitude is around twice as fast as the subduction velocity in the SOP model (3 cm/year vs 1.5 cm/year), resulting in a timing for the breakoff that is around twice shorter in the WOP model than in the SOP model (12 My vs 21 My).

**Figure 11:**
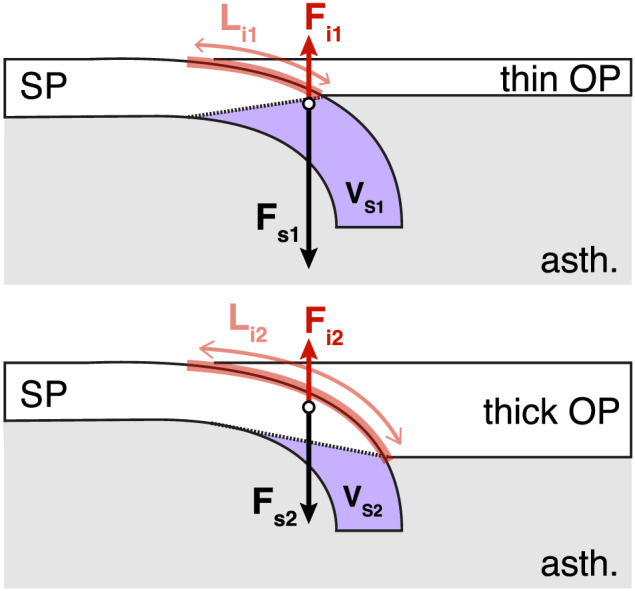
Sketch illustrating the difference between slab pull force (*F*_s_) and force at the subduction interface (*F*_i_) depending on the overriding plate (OP) thickness (thin on top and thick at the bottom). *L*_i_ corresponds to the length of the subduction interface, and *V*_s_ is the volume of slab considered (see text for details).

## 6 Discussion

### 6.1 Multiple Effects of Changing OP Thickness

The parametric study presented here shows that despite keeping all the parameters of our models constant, apart from the thickness/strength of the OP, our subduction/collision system shows first‐order differences in terms of geometry, strain, and kinematics. Consequently, these differences may in turn help gaining insights into the past rheological nature of the overriding lithosphere in natural systems. The effect of a change in lithospheric thickness is threefold:

1. An initially thinner and hotter OP has a lower strength, which, under the same amount of stress, favors OP stretching and associated trench retreat. During the first stages of the model, the trench retreat is twice as fast in the WOP model as in the SOP model (~4 cm/year vs ~2 cm/year, Figure 3). As a result, slab rollback is favored and, considering that the absolute motion of the SP is identical in all models, the subduction velocity should, on average, be higher in the weakest models.
2. The thickness of the OP also controls the space available for sublithospheric mantle to flow beneath the OP. As a result, within the first stages of the model, the magnitude of the poloidal flow that develops above the slab for a thick lithosphere is small and only affects the leading edge of the slab. Instead, in models with a thin OP, mantle flow associated with slab sinking reaches higher magnitudes and affects a larger segment of the slab length, promoting slab rollback (Figure 2). This is in agreement with previous studies that show that the mantle flow in the mantle wedge can be affected by a thickened OP (e.g., O'Driscoll et al., 2009) and that the thickness of OP affects the subduction style by modifying the slab dip angle and trench motions (e.g., Li et al., 2019). In subsequent stages of subduction, when the mode of subduction is established, the presence or absence of a tail at the 660‐km discontinuity under the OP may also affect the mantle circulation. It would further constrain the space available for the mantle to flow, which in turn could alter the dynamics of subduction by modifying the dynamic pressure above the slab (Holt et al., 2017).
3. The thickness of the OP controls the length of the plate interface and therefore the coupling between plates. Following the approach of Martinod et al. (2010), the shear force within the subduction interface may be written as

(19) $$F_{i}=\frac{\eta_{i}v_{i}L_{i}}{D_{i}}.$$

This equation states that the force at the interface *F*_*i*_ is proportional to *η*_*i*_, *v*_*i*_, *L*_*i*_, and *D*_*i*_ (corresponding to the viscosity at the interface, the shear velocity, the length, and the thickness of the interface, respectively). Following this equation, if the length of the interface (*L*_*i*_) increases, the shear force at the interface *F*_*i*_ increases. This is true if all the other parameters (*η*_*i*_, *v*_*i*_, and *D*_*i*_) remain constant with time and from one configuration to the other. However, looking only at the force at the interface is not sufficient with our set of models because other forces, in particular slab pull, also change when changing OP thickness. Indeed, if the thickness of the lithosphere increases, *L*_*i*_ increases but at the same time the slab pull force is reduced because the surrounding asthenosphere on the side of the OP is replaced by colder and denser lithosphere (Figure 11).

We therefore now compare the force at the plate interface and the slab pull force for the two end‐member models SOP and WOP at the beginning of the simulations, which is fundamental in the further evolution of the models.

For this, the second invariant of the stress field was extracted at the interface and integrated over the entire surface of the subduction plane interface and used to compute the total force as

(20) $$F_{i}=\sigma_{\mathrm{II}}d_{y}L_{i}$$

where *σ*_II_ is the second invariant of the stress field at the interface, *d*_*y*_ is the width of the interface cell in the *y* direction, and *L*_*i*_ is the length of the interface. In our models, as the interface is simulated as a weak zone, the value of *σ*_II_ is low, and therefore the force at the interface is small and only slightly varies between models (between 1.32 × 10^17^ and 1.18 × 10^17^ N). The force per length unit of the interface (*F*_*i*_/*L*_*i*_ in N/m) is then higher for shorter interface length (*F*_i1_ > *F*_i2_ for *L*_i1_ < *L*_i2_, see Figure 11). We obtain values of 9.94 × 10^11^ N/m in the WOP model and 4.92 × 10^11^ N/m in the SOP model.

The slab pull force *F*_*s*_ is computed from our models, following the following equation:

(21) $$F_{s}=∆\rho V_{s}g$$

where *Δρ* corresponds to the density difference between the slab (averaged from our models over the part of the mesh constituting the slab volume) and the asthenosphere (constant using equation (7) with *T* = *T*_asth_), *V*_s_ is the volume of slab, and *g* is the acceleration of gravity.

Since the thickness of the SP is the same for all models at the initial stage, the slab pull force (*F*_s_) is calculated by only taking the slab volume in the asthenosphere (Figure 11). Hence, the thicker the OP is, the lower the slab pull force is (*F*_s2_ < *F*_s1_, see figure). We obtain values for the slab pull force that, once divided by the subduction interface length, has a value of 3.03 × 10^12^ N/m in the WOP model (larger slab volume in the asthenosphere) and of 1.13 × 10^12^ N/m for the SOP model (lower slab volume in the asthenosphere).

These results show the following:

1. The forces at the interface are comparable regardless of the length of the interface. The average value of *σ*_II_ is on the order of 25–50 MPa (53.5 MPa in the WOP model and 26.5 MPa in the SOP model), comparable to values proposed in previous studies (e.g., Duarte et al., 2015; Lamb and Davies, 2003).
2. Due to the thickness of the OP, the slab pull forces at the beginning of the model are very different (~ 2.7 times higher in the WOP model than in the SOP model).
3. The coupling force at the interface is always lower than the slab pull force, which explains the fact that subduction always occurs. However, the force ratio between *F*_s_ and *F*_i_ decreases when the thickness of the OP increases. The slab pull force is ~3.0 times higher than the coupling force at the interface in the WOP model and ~2.3 times higher in the SOP model. This could explain why slab roll back is promoted in cases involving thin/weak OPs where the force allowing the slab to move back is higher.

### 6.2 Folding Mode Versus Rollback Mode

The switch between folding mode and rollback mode occurs for lithospheric thickness value of about 150 km (Figure 9). This is due to the initial force balance in the system that controls the geometry of the slab when it arrives at the 660‐km discontinuity, which in turn determines the mode of subduction and the subsequent evolution of the model. This binary mode is probably due to the fact that above a certain OP thickness (here ~ 150 km), a threshold is reached for the three mechanisms described above. However, deciphering the relative contribution of each of these mechanisms is beyond the scope of this study. Tomographic images of slabs at depth in natural cases could therefore give us, in association with other key parameters (see below), information on the past rheological characteristics of the OP. This value of 150 km needs however to be taken with caution as it depends on the amount of coupling between the plates and on the initial geometry chosen here for the slab (initial slab length of 200 km with a curvature radius of 500 km), which may influence the mode of subduction as the length of the plates’ interface depends on slab dip. In addition, the imposed initial thermal profile for the lithosphere does not take into account the subduction processes and associated mantle flow involved to reach this initial configuration. To reach this geometry, the lithospheric mantle above the mantle wedge can be thermally eroded at the base of the lithosphere at a faster rate than the diffusion rate. Hence, this value of 150 km rather corresponds to an upper bound value. Moreover, the thickness/strength of the OP is parameterized here with a prescribed thermal profile, but in nature there are many potential reasons for such changes in the thickness, including the nature of the OP, subduction erosion, underplating, dehydration of slabs, and magmatism. These points deserve more attention in future studies.

### 6.3 Variability in Time of the Tectonic Processes

The thickness of the OP also has a strong influence on the velocities and on the timing of the different events at convergent margins. The stresses required to deform, at the same rate, a strong OP and an associated long subduction interface are larger than those required to deform a weak OP and a short subduction interface. The only engine of our system is the slab pull, which only slightly changes between models as a function of slab geometry. Thus, the remaining energy that would be left to deform the mantle or to displace the plates is lower for the SOP models. This explains why the subduction velocity is lower with a SOP than with a WOP. It requires more time for the OP to deform and open back‐arc basins at the latitude of the oceanic subduction. We also observe that the depth at which slabs break off is independent from the initial thickness of the OP, occurring at around 300 km, which is within the range of breakoff depths proposed in previous studies (40 to over 500 km; Baumann et al., 2010; Duretz et al., 2011). As a consequence, with the subduction velocity being lower in the SOP models, it requires a longer time for the continental block to reach such depths where the positive buoyancy of the continental block triggers slab necking and eventually breakoff. Moreover, after slab breakoff, a weak OP allows the eduction of the crustal material while with a strong one this same crustal material stays at deeper levels. With our setup, the variability in the timing of these processes is quite large: it takes from ~5 to ~25 My after continental subduction initiation for back‐arc basins to form and from ~10 to ~30 My for slabs to break off. For very strong OP, back‐arc basins may not even form within the OP. These values for the time of back‐arc rifting after collision are in the range of what can be observed in natural example (e.g., see Table 1 in Wallace et al., 2009). The occurrence and timing of these different events (continental subduction, back‐arc basins formation, and slab breakoff) in natural systems could therefore also help, to a certain extent, in constraining the past rheological characteristics of the OP.

### 6.4 Deformation Above the Subducting Plate

By controlling the plates interface length, the subduction velocity, the trench velocity, the slab dip, and the mantle flow pattern, the rheological properties of the OP influence the trench shape and the OP deformation pattern. A stronger OP provides a higher resistance to extension and curving, in agreement with Meyer et al. (2013). In addition, after continental subduction, the upper plate maintains deformation by shortening around the continental block for a long period of time (over 20 My), in agreement with Butterworth et al. (2012), while it displays a synchronous combination of shortening and stretching on a shorter time period (12 My) with a weak OP. This explains high topography in front of the strong OP, whereas the weak OP displays lower and more diffuse topography. Such variability (or absence of variability) in the style of deformation and topography may be extracted from the geological record and/or from present‐day observations and could also give information on the past rheological characteristics of the OP. Concerning the topographic signal, only relative variations may be considered here as the elevation is indirectly inferred from the vertical stresses at the surface of the model and does not take into account erosion/deposition processes. Our model has some limitations regarding the rheological parameters used. As these parameters for natural rocks are poorly constrained, we represent them with typical flow laws (see section 2.1). Modifying the parameters of these flow laws or considering different rheological approximations for the lithosphere (e.g., linearly viscous, stratified nonlinear temperature‐dependent visco‐elasto‐plastic rheology) may lead to changes in the geometry of the system as well as in the timing and location of the deformation, as discussed, among others, by Pusok et al., (2018).

## 7 Conclusions

The key result of this study is therefore that the thickness/strength of the OP, in particular near the trench, has a strong influence on subduction and collision dynamics. Thin/weak OP promotes slab rollback, more intense mantle flow above the slab, and faster trench migration compared to a thick/strong OP. The thickness/strength of the OP also controls the timing of the subduction zone dynamics. Slab breakoff and back‐arc basin formation occur significantly earlier in models involving a thin OP. Finally, because an increase of the OP thickness leads to an increase of the subduction interface length and a decrease of the slab pull force, consequences on the coupling between plates can be important. Future studies focused on the nature of this interface (considered weak in this study), in nature and models, are thus an interesting avenue for further research in subduction zones dynamics.
